# Adjuvant Chemotherapy for Resected Stage-IA Lung Adenocarcinoma With Micropapillary Histologic Pattern

**DOI:** 10.1093/icvts/ivag165

**Published:** 2026-06-09

**Authors:** Yifei Zhou, Dongliang Bian, Fujun Yang, Xinnan Xu, Yingran Shen, Linsong Chen, Jie Dai, Xiaoxiong Xu, Yuming Zhu, Deping Zhao, Peng Zhang, Xiaogang Liu, Kaiqi Jin, Gening Jiang

**Affiliations:** Department of Thoracic Surgery, Shanghai Pulmonary Hospital, School of Medicine, Tongji University, Shanghai 200433, China; Department of Thoracic Surgery, Shanghai Pulmonary Hospital, School of Medicine, Tongji University, Shanghai 200433, China; Department of Thoracic Surgery, Shanghai Pulmonary Hospital, School of Medicine, Tongji University, Shanghai 200433, China; Department of Thoracic Surgery, Shanghai Pulmonary Hospital, School of Medicine, Tongji University, Shanghai 200433, China; Department of Thoracic Surgery, Shanghai Pulmonary Hospital, School of Medicine, Tongji University, Shanghai 200433, China; Department of Thoracic Surgery, Shanghai Pulmonary Hospital, School of Medicine, Tongji University, Shanghai 200433, China; Department of Thoracic Surgery, Shanghai Pulmonary Hospital, School of Medicine, Tongji University, Shanghai 200433, China; Department of Thoracic Surgery, Shanghai Pulmonary Hospital, School of Medicine, Tongji University, Shanghai 200433, China; Department of Thoracic Surgery, Shanghai Pulmonary Hospital, School of Medicine, Tongji University, Shanghai 200433, China; Department of Thoracic Surgery, Shanghai Pulmonary Hospital, School of Medicine, Tongji University, Shanghai 200433, China; Department of Thoracic Surgery, Shanghai Pulmonary Hospital, School of Medicine, Tongji University, Shanghai 200433, China; Department of Thoracic Surgery, Shanghai Pulmonary Hospital, School of Medicine, Tongji University, Shanghai 200433, China; Department of Thoracic Surgery, Shanghai Pulmonary Hospital, School of Medicine, Tongji University, Shanghai 200433, China; Department of Thoracic Surgery, Shanghai Pulmonary Hospital, School of Medicine, Tongji University, Shanghai 200433, China

**Keywords:** lung adenocarcinoma, stage IA, micropapillary pattern, resection, adjuvant chemotherapy

## Abstract

**Objectives:**

The micropapillary pattern (MP) is a high-risk factor for postoperative recurrence in lung adenocarcinoma (LUAD), but the role of adjuvant chemotherapy (ACT) in stage IA LUAD with MP (LUADmp) after resection remains unclear.

**Methods:**

From September 2017 to August 2020, in the phase II trial, resected stage IA LUADmp patients received ACT (pemetrexed and carboplatin), forming the ACT-group. Patients who met the trial criteria but declining ACT during the same period were designated as the standard of care (SoC)-group. Recurrence-free survival (RFS) was compared between groups using univariate and multivariate analyses.

**Results:**

The numbers of patients in ACT-group and SoC-group were 81 and 75, respectively. Compared to SoC-group, patients in ACT-group were younger (60.0 vs 64.0 years, *P *= .019), had a lower proportion of EGFR/ALK mutations (58.0% vs 78.7%, *P *= .006), and had a higher proportion of spread through air spaces (59.3% vs 40.0%, *P *= .016). At the cut-off date of January 31, 2025, the median duration of follow-up was 65.6 months. Five-year RFS in ACT-group met the prespecified primary end-point, and no significant difference was observed between ACT-group and SoC-group (ACT-group vs SoC-group: 88.9% vs 86.7%, log-rank *P* = .534). Multivariate analysis showed ACT could not predict RFS independently (hazards ratio [HR], 0.865, 95% CI, 0.334-2.240, *P *= .765).

**Conclusions:**

Adjuvant chemotherapy was not significantly associated with improved RFS for stage IA LUADmp patients postoperatively. This study was registered at ClinicalTrials.gov: NCT03351842.

**Clinical registration number:**

ClinicalTrials.gov Identifier: NCT03351842.

## Graphical abstract

**Figure:**
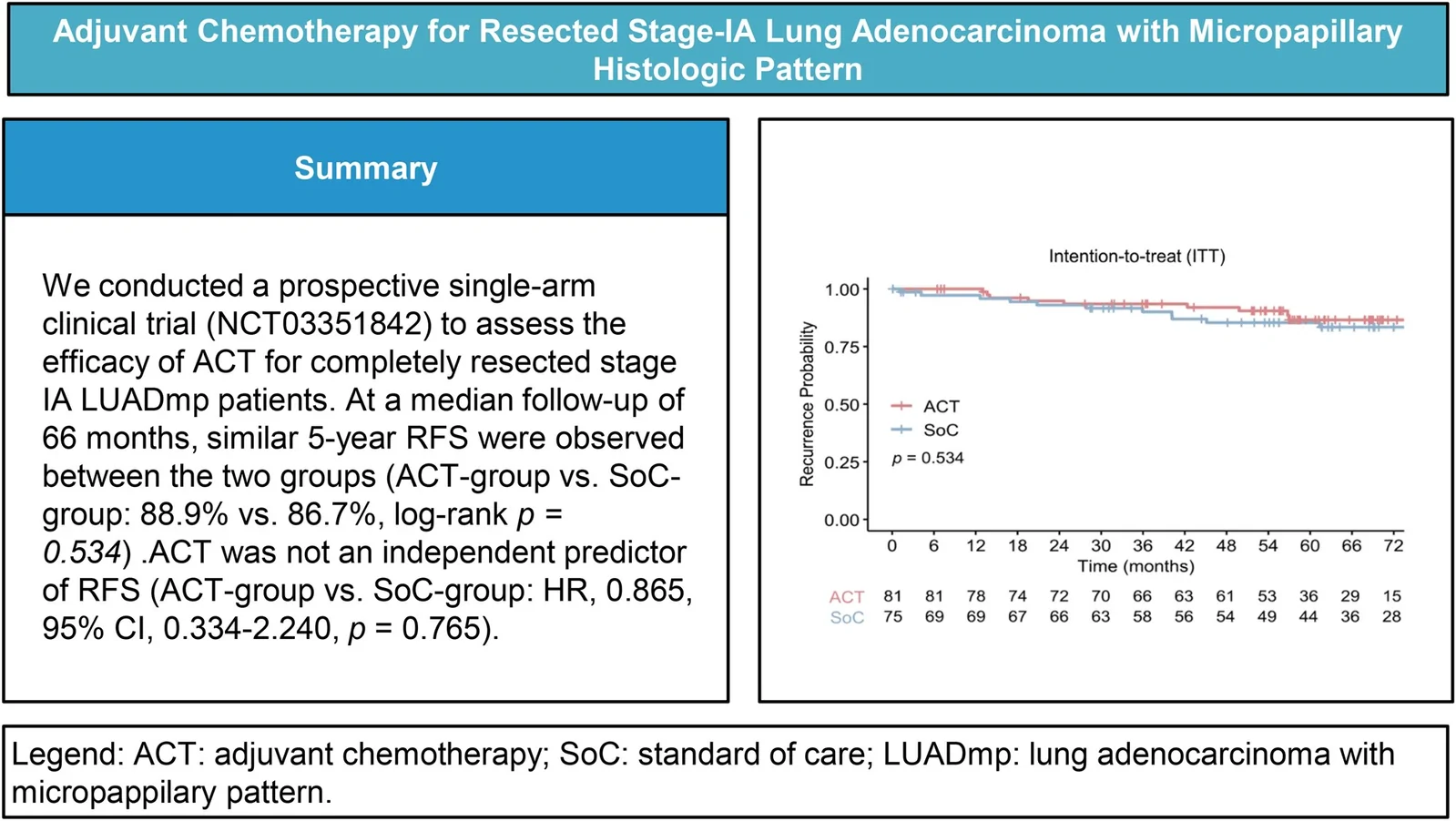

## INTRODUCTION

Anatomical pulmonary resection is a standard therapy for stage I non-small cell lung cancer (NSCLC). Approximately 30% of stage I NSCLC patients die postoperatively mainly due to tumour recurrence.^1^ Because of the high rate of recurrence and mortality, surgery alone may not be sufficient for stage I NSCLC. Resection followed by adjuvant chemotherapy (ACT) has been recommended as the standard therapy for stage IB NSCLC with high-risk factors of recurrence.^2–8^ Nevertheless, the 5-year recurrence-free survival (RFS) rate for NSCLC patients in stage IA was lower than 90% postoperatively, especially for patients with high-risk factors for recurrence.^2^^,^^9^

Micropapillary pattern (MP) was defined in the new classification for lung adenocarcinoma (LUAD) in 2005^10^ and was identified as a high-risk factor of recurrence for LUAD. Lung adenocarcinoma patients with MP (LUADmp) had a significantly higher recurrence rate than those without,^8^^,^^10–13^ even among patients with stage IA disease (with MP vs without MP: 5-year RFS, 69.7% vs 89.3%, *P *< .001).^13^ In clinical practice, under the premise of controlling the side effects, a few of stage IA LUAD patients were recommended to receive ACT if they were with high-risk factors of recurrence, even though no evidence support these patients could benefit from ACT.

As of now, clinical trials concerning resection followed by ACT for stage IA LUAD patients are relatively scarce. In 2006, Nakagawa et al^5^^,^^14^ conducted a randomized phase 3 trial, which concluded that ACT may be effective for surgically resected LUAD patients with pathological stage I disease (ACT vs standard of care [SoC]: 5-year overall survival [OS], 83.6% vs 77.9%, *P *= .017). In 2020, Pathak et al^15^ demonstrated that the prognosis of stage IA NSCLC was not improved with ACT (ACT vs SoC: OS, hazards ratio [HR] = 1.10, 95% CI, 0.96-1.26, *P *= .17). These studies, however, did not stratify by risk factors for stage IA patients. The efficacy of ACT for stage IA LUADmp remains unclear.

This study assessed the efficacy of ACT postoperatively in resected stage IA LUADmp patients.

## METHODS

### Study design

#### Selection and description of participants

A prospective, single-center, phase II clinical trial enrolled participants who presented to Shanghai Pulmonary Hospital between September 1, 2017 and August 31, 2020 and met the following criteria: (1) patients with pathological stage IA LUAD (pT1a-cN0M0), (2) patients undergoing lobectomy or segmentectomy in combination with lymph node dissection, (3) patients with histopathological MP pattern, and (4) patients receiving no anti-cancer treatment before surgery. The exclusion criteria were: (1) age > 75 years, (2) patients with a history of cancer within 5 years, (3) patients who did not undergo an anatomic resection in combination with lymph node dissection, and (4) interval between surgery and the first dose of ACT beyond 3 months. Segmentectomy was only performed for patients with poor pulmonary reserve or other major comorbidities that contraindicated lobectomy. Participants who were enrolled in this trial were classified as ACT-group. In addition, we selected patients who met the criteria but refused to enrol in this trial in our institution in the same time period and were retrospectively recorded as observation group (SoC-group) to evaluate the role of ACT for stage IA LUADmp patients. Lung adenocarcinoma patients with MP patients were defined as those whose tumours contain an MP component. And these patients were further divided into 2 subgroups according to the immunohistochemical (IHC) results of resected LUAD tissue: the MP++ group if the MP was predominant and the MP+ group if micropapillary components were secondary predominant. All patients included in the analysis met these strict pathological criteria, as confirmed by 2 independent pathologists using standardized IASLC criteria.^16^ This study was registered with ClinicalTrials.gov (NCT03351842), and the protocol and statistical analysis plan can be accessed in the supplement material.

#### Ethics

This study was approved by the Shanghai Pulmonary Hospital independent ethics committee (ID: K17-151-3). Written informed consent was obtained from all patients in the ACT-group (patients who were enrolled in our clinical trial) and was waived in the SoC-group (patients who were retrospectively recorded). The collection and storage of data or biological material from research participants for multiple and indefinite use was consistent with requirements outlined in the WMA Declaration of Taipei.

### Technical information

#### Procedures

Patients in ACT-group received the following drugs intravenously as adjuvant treatment: pemetrexed (500 mg/m^2^) combined with carboplatin (area under the curve 5), on day 1 of each 21-day cycle, for 4 cycles postoperatively.

Outpatient follow-up was scheduled every 3 months over the first postoperative year, then every 6 months for 2 to 5 years, and annually thereafter. Follow-up data including recurrence and survival status were updated by telephone contact annually. Recurrence was diagnosed by CT, PET/CT, brain MRI, and/or tissue biopsy.

#### Efficacy assessment

The primary end-point of the phase II trial was 5-year RFS in ACT-group. The secondary end-points of this trial were 5-year OS and treatment-related adverse event (TRAEs).

#### Sensitivity analysis

To further assess the robustness of the primary findings and address potential baseline imbalance between the ACT and SoC groups, a propensity score-based sensitivity analysis was performed. Propensity scores were estimated using a logistic regression model incorporating clinically relevant covariates that could influence treatment selection and recurrence risk, including age, gender, MP proportion, EGFR/ALK mutation status, lymphatic-vascular invasion (LVI), spread through air spaces (STAS), and surgical procedure.

In addition, an extended multivariable Cox proportional hazards model was constructed as a sensitivity analysis by further adjusting for surgical margin distance, modelled as a continuous variable, to explore the potential confounding impact of surgical factors on RFS. These analyses were prespecified as sensitivity analyses and were not intended to replace the primary efficacy assessment of the prospective ACT cohort.

### Statistics

Categorical and continuous variables were compared between the ACT-group and SoC-group by the chi-square test and Student’s *t*-test tor the Mann-Whitney *U*-test (for non-normally distributed data), respectively. Differences in RFS between the 2 groups were compared by the Kaplan-Meier method and log-rank test. Recurrence-free survival was defined as the interval from the date of surgery to recurrence or censorship. Recurrence-free survival for censored patients was defined as the date of last telephone contact or last outpatient visit, whichever was most recent. Multivariate survival analysis was calculated by Cox proportional hazards modelling. Statistical analyses were performed by SPSS Statistics 26 (IBM Corp.). Two-tailed *P* value < .05 was considered to indicate statistical significance. *P* value were unadjusted for multiplicity. We did not use any AI tool to complete the article.

## RESULTS

### Characteristics of patients

A total of 156 patients enrolled in the study, with 81 in the ACT-group, and 75 in the SoC-group (**Figure S1**). Clinical characteristics of patients are listed in **Table 1**. The median age at surgery was 61.5 years (range, 31-75 years), median pathological maximum tumour dimension (pMTD) was 20.0 mm (range, 5.0-30.0 mm), median surgical margins was 3 cm (range, 0.5-7), median lymph node dissections was 10.0 (range, 1-27), median number of lymph node dissection stations was 6 (range, 1-9), and median MP proportion of the tumour was 15.0% (range, 5%-100%). The majority were female (53.8%), never-smokers (79.5%), harbouring *EGFR*/*ALK* mutations (67.9%), and receiving lobectomy (85.9%). Most patients received systematic lymphadenectomy, except 2 (1.3%) patients received sampling lymphadenectomy, and 5 (3.2%) patients received lobe-specific lymphadenectomy. All patients received R0 resection. The median age of patients in the ACT-group was significantly younger than those in the SoC-group (60.0 vs 64.0 years, *P *= .019). Significant differences in the proportion of patients with *EGFR*/*ALK* mutations and STAS were observed between the 2 groups (ACT-group vs SoC-group: positive *EGFR*/*ALK* mutation, 58.0% vs 78.7%, *P *= .006; positive STAS, 59.3% vs 40.0%, *P *= .016). No significant difference regarding other variables was observed between these 2 groups. In the ACT-group, 64 (79.0%) patients received ACT for 4 cycles as scheduled without cycle delay or dosage reductions. The remaining 17 (21.0%) patients did not complete the schedule due to serious adverse events (SAEs) (3 patients with neutropenia, 2 patients with increased aminotransferases, and 1 patient with increased creatinine) or other personal reasons (11 patients refused to continue ACT). All patients in the SoC group did not receive any anti-cancer treatment after surgery.

**Table 1.** Baseline Characteristics of Resected NSCLC Patients with Stage IA Disease and MP Pattern

Variables	No. of patients (%)			*P*
	All	ACT	SoC	
	(*N* = 156)	(*N* = 81)	(*N* = 75)	
Median age, years (range)	61.5 (31-75)	60 (31-74)	64 (38-75)	.019
Gender, number (%)				.604
Male	72 (46.2)	39 (48.1)	33 (44.0)	
Female	84 (53.8)	42 (51.9)	42 (56.0)	
Smoking status, number (%)				.807
Ever	32 (20.5)	16 (19.8)	16 (21.3)	
Never	124 (79.5)	65 (80.2)	59 (78.7)	
EGFR/ALK mutation, number (%)				.006
Positive	106 (67.9)	47 (58.0)	59 (78.7)	
Negative or unknown	50 (32.1)	34 (42.0)	16 (21.3)	
Surgical procedure, number (%)				.115
Lobectomy	134 (85.9)	73 (90.1)	61 (81.3)	
Segmentectomy	22 (14.1)	8 (9.9)	14 (18.7)	
pMTD, mm (range)	20.0 (5-30)	18.0 (8-30)	20.0 (5-30)	.812
LND, No. (range)	10.0 (1-27)	10.5 (1-24)	10.2 (1-27)	.739
Surgical margins, cm (range)	3.0 (0.5-7)	3.0 (1-7)	3.0 (0.5-7)	.083
LNDS, No, (range)	6 (1-9)	6 (2-8)	6 (1-9)	.332
Histological grade				.053
Grade 2	76 (48.7)	46 (56.8)	30 (40.0)	
Grade 3	80 (51.3)	35 (43.2)	45 (60.0)	
LVI, number (%)				.214
Positive	11 (7.1)	8 (9.9)	3 (4.0)	
Negative	145 (92.9)	73 (90.1)	72 (96.0)	
STAS, number (%)				.016
Positive	78 (50.0)	48 (59.3)	30 (40.0)	
Negative	78 (50.0)	33 (40.7)	45 (60.0)	
MP proportion, % (range)	15.0 (5-100)	10.0 (5-100)	20.0 (5-80)	.548
MP pattern, number (%)				.929
Predominant (MP++)	17 (10.9)	9 (11.1)	8 (10.7)	
Secondary (MP+)	139 (89.1)	72 (88.9)	67 (89.3)	
Prognosis, number (%)				.671
Recurrence	20 (12.8)	9 (11.1)	11 (14.6)	
Non-recurrence	136 (87.2)	72 (88.9)	64 (85.3)	
Prognosis at 5-years, number (%)				.858
Recurrence	19 (12.2)	9 (11.1)	10 (13.3)	
Non-recurrence	137 (87.8)	72 (88.9)	65 (86.7)	

Abbreviations: ACT = adjuvant chemotherapy; LND = lymph node dissection; LNDS = lymph node dissection station; LVI = lymphatic-vascular invasion; MP = micropapillary pattern; NSCLC = non-small cell lung cancer; pMTD = pathological maximum tumour dimension; SoC = standard of care; STAS = spread through air spaces.

### Survival analyses

At the cutoff date of January 31, 2025, the median duration of follow-up was 65.6 months (interquartile range [IQR], 53.7-74.6 months). Thirty-four patients were censored during the follow-up period, with a loss to follow-up rate of 21.8%. In addition, in ACT-group and SoC-group, there were 9 and 11 patients who experienced recurrence, respectively (11.1% vs 14.6%, *P *= .671). Four patient deaths were recorded during follow-up, with all cases occurring in the SoC-group and were all associated with cancer recurrence. Between ACT-group and SoC-group, no significant differences in RFS were observed at 12 months (100.0% vs 97.3%, *P *= .230), 36 months (93.8% vs 90.7%, *P *= .554), and 60 months (88.9% vs 86.7%, log-rank *P* = .534, **Figure 1A**), respectively. The 5-year RFS rate in ACT-group met the prespecified primary end-point of this trial. Multivariate survival analysis demonstrated that RFS of pathological stage IA LUADmp was not influenced by ACT treatment (ACT-group vs SoC-group: HR, 0.865, 95% CI, 0.334-2.240, *P *= .765). In addition, RFS was not influenced by each variable (**Figure 2A-D**), including the proportion of MP (MP predominant [MP++] vs MP secondary [MP+] subgroups: log-rank *P *= .572). Multivariate survival analysis demonstrated that RFS was not influenced by the proportion of MP pattern for pathological stage IA LUADmp (MP+ vs MP++: HR, 0.485, 95% CI, 0.113-2.090, *P *= .332) (**Table 2**).

**Figure 1.**
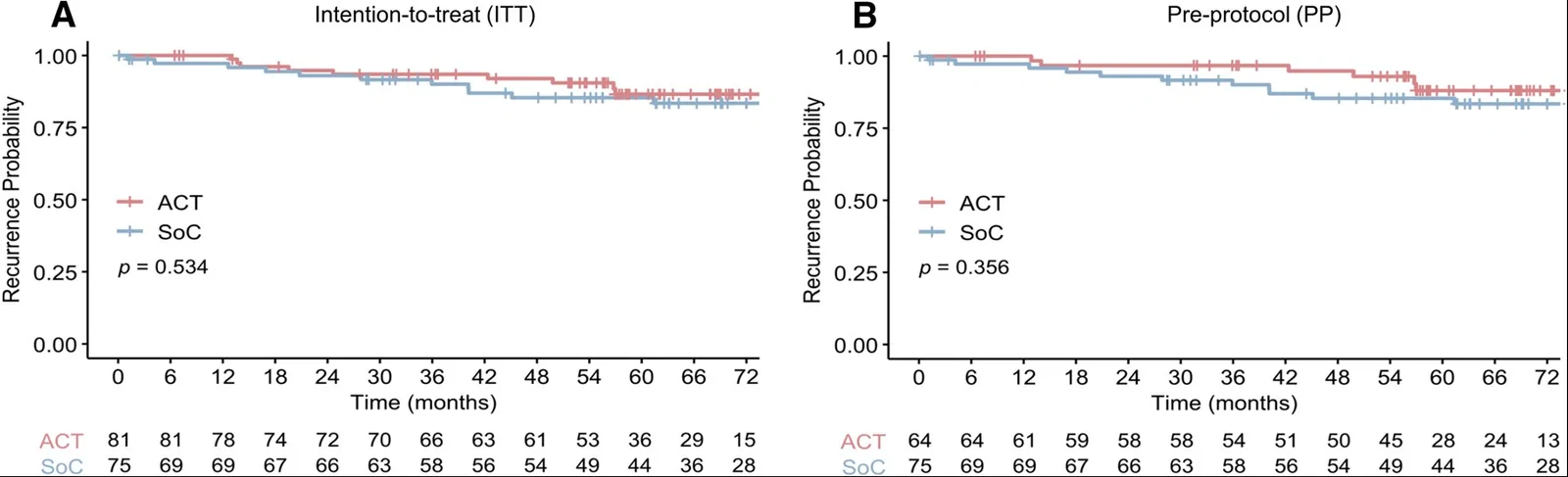
Recurrence-Free Survival among Patients. (A) Recurrence-free survival curve among the entire cohort of resected stage I LUADmp patients (*N* = 156). There was no significant difference in recurrence-free survival between patients receiving any adjuvant chemotherapy (ACT, *N* = 81) and standard of care (SoC, *N* = 75) (5-year RFS: 88.9% vs 86.7%, log-rank *P *= .534). (B) Recurrence-free survival curve among patients, excluding those in the ACT-group who did not complete the full 4 cycles of treatment. There was no significant difference in recurrence-free survival between patients receiving a full 4 cycles of adjuvant chemotherapy (ACT, *N* = 64) and standard of care (SoC, *N* = 75) (5-year RFS: 90.6% vs 86.7%, log-rank *P *= .356).

**Figure 2.**
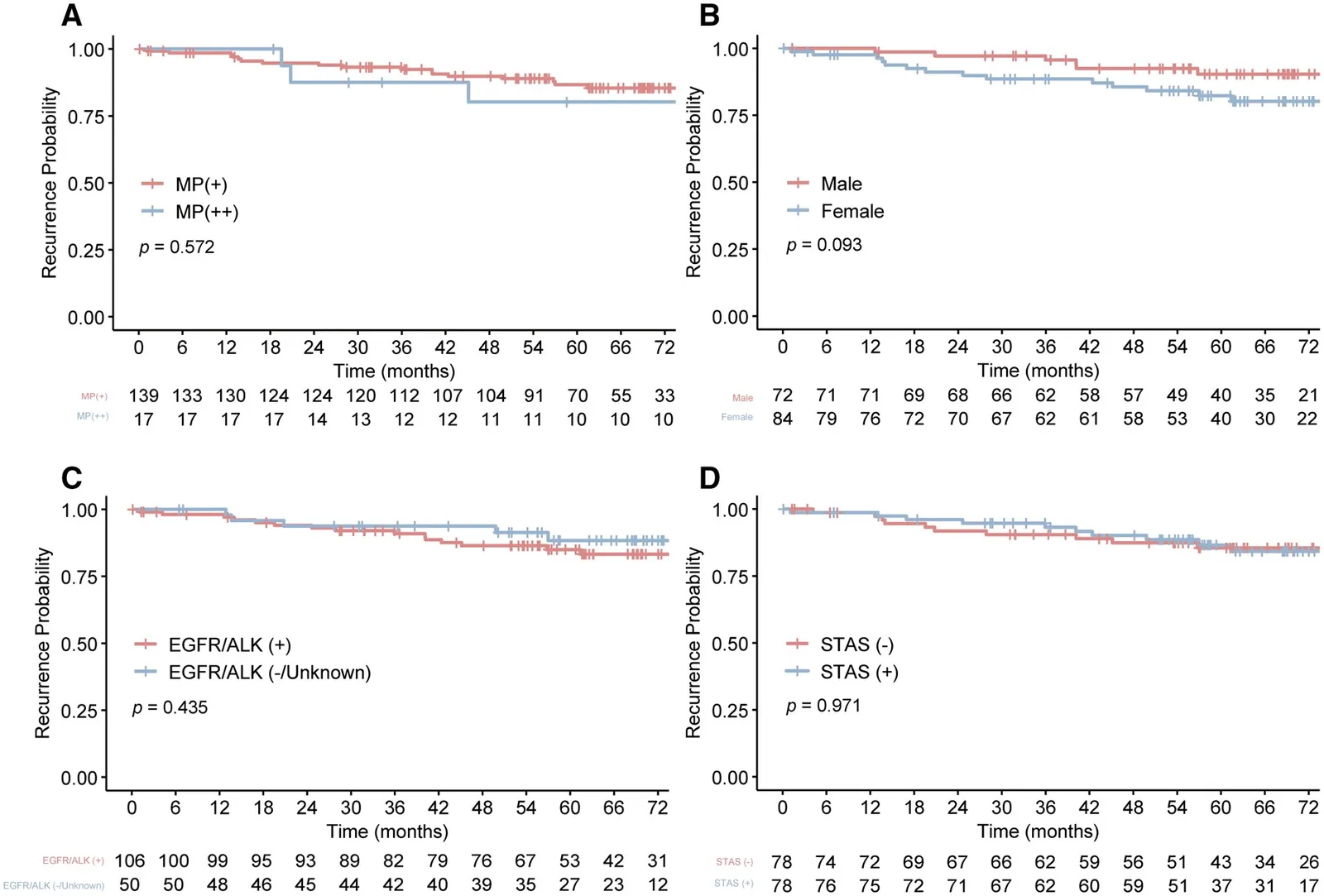
Recurrence-Free Survival among Patients between Each Variable. (A) Recurrence-free survival between the MP++ and MP+ patients (log-rank *P* = .572). (B) Recurrence-free survival between male and female patients (log-rank *P* = .093). (C) Recurrence-free survival between EGFR/ALK mutation and EGFR/ALK wild-type or unknown patients (log-rank *P* = .435). (D) Recurrence-free survival between STAS positive and STAS negative patients (log-rank *P* = .971). Abbreviations: MP = micropapillary pattern; STAS = spread through air spaces.

**Table 2.** Univariate and Multivariate Survival Analysis by Cox Proportional Hazards Modelling Including All Study Patients (*n* = 156)

Variables	Univariate			Multivariate		
	HR	95% CI	*P*	HR	95% CI	*P*
Age (years)						
≤60	1.000			1.000		
>60	1.482	0.591-3.717	.401	1.711	0.660-4.433	.269
Gender						
Male	1.000			1.000		
Female	2.221	0.854-5.718	.102	5.482	1.093-27.499	**.039**
Smoking status						
Ever	1.000			1.000		
Never	0.830	0.301-2.283	.717	0.264	0.048-1.462	.127
EGFR/ALK mutation						
Positive	1.000			1.000		
Negative or unknown	0.670	0.244-1.844	.438	0.747	0.261-2.135	.586
Surgical procedure						
Lobectomy	1.000			1.000		
Segmentectomy	0.619	0.144-2.674	.521	0.671	0.141-3.200	.616
LVI						
Positive	1.000			1.000		
Negative	1.377	0.184-10.300	.521	3.428	0.302-38.912	.320
STAS						
Positive	1.000			1.000		
Negative	1.017	0.409-2.363	.971	0.744	0.291-1.898	.536
MP Pattern						
Predominant (MP++)	1.000			1.000		
Secondary (MP+)	0.703	0.206-2.400	.574	0.485	0.113-2.090	.332
Adjuvant treatment						
SoC	1.000			1.000		
ACT	0.756	0.313-1.827	.535	0.865	0.334-2.240	.765

Abbreviations: ACT = adjuvant chemotherapy; CI = confidence interval; HR = hazards ratio; LVI = lymphatic-vascular invasion; MP = micropapillary pattern; SoC = standard of care; STAS = spread through air spaces.

Excluding patients in ACT-group who completed less than 4 cycles of chemotherapy (*n* = 17), the remaining 139 patients formed the “per-protocol” set. As **Table S1** indicated, no high-risk factors for recurrence were identified by multivariate analyses for this set. In addition, RFS were similar between patients in the ACT-group and the SoC-group (ACT-group vs SoC-group: HR, 0.643, 95% CI, 0.225-1.838, log rank *P* = .356) (**Figure 1B**).

Furthermore, a sensitivity analysis was conducted to assess the robustness of the primary findings. We performed PSM sensitivity analysis and found that ACT remained not significantly associated with improved RFS (HR: 1.821, 95% CI, 0.593-5.591, *P* = .295, **Figure S2**). The baseline characteristics after PSM were shown in the **Table S2.** 

### Subgroup analysis for RFS

Subgroup analyses for RFS are shown in **Table 3** and **Figure 3**. For resected pathological stage IA LUADmp, no significant differences in 5-year RFS between the ACT-group and the SoC-group were observed among all subgroups. Regardless of the proportion of MP pattern, there was no significant difference in RFS at 5 years between patients receiving ACT or not (MP++ subgroup: 88.9% vs 75.0%, *P *= .600; MP+ subgroup: 88.9% vs 88.1%, *P *= .690). Furthermore, we recognized that Stage IA spans IA1-IA3, representing distinct biological subsets with different recurrence risks. Therefore, we compared the survival outcomes among patients with IA1, IA2, and IA3 disease and found statistically significant differences (**Figure S3A**). However, within each substage (IA1, IA2, or IA3), ACT was not associated with improved survival (**Figure S3B-D**).

**Figure 3.**
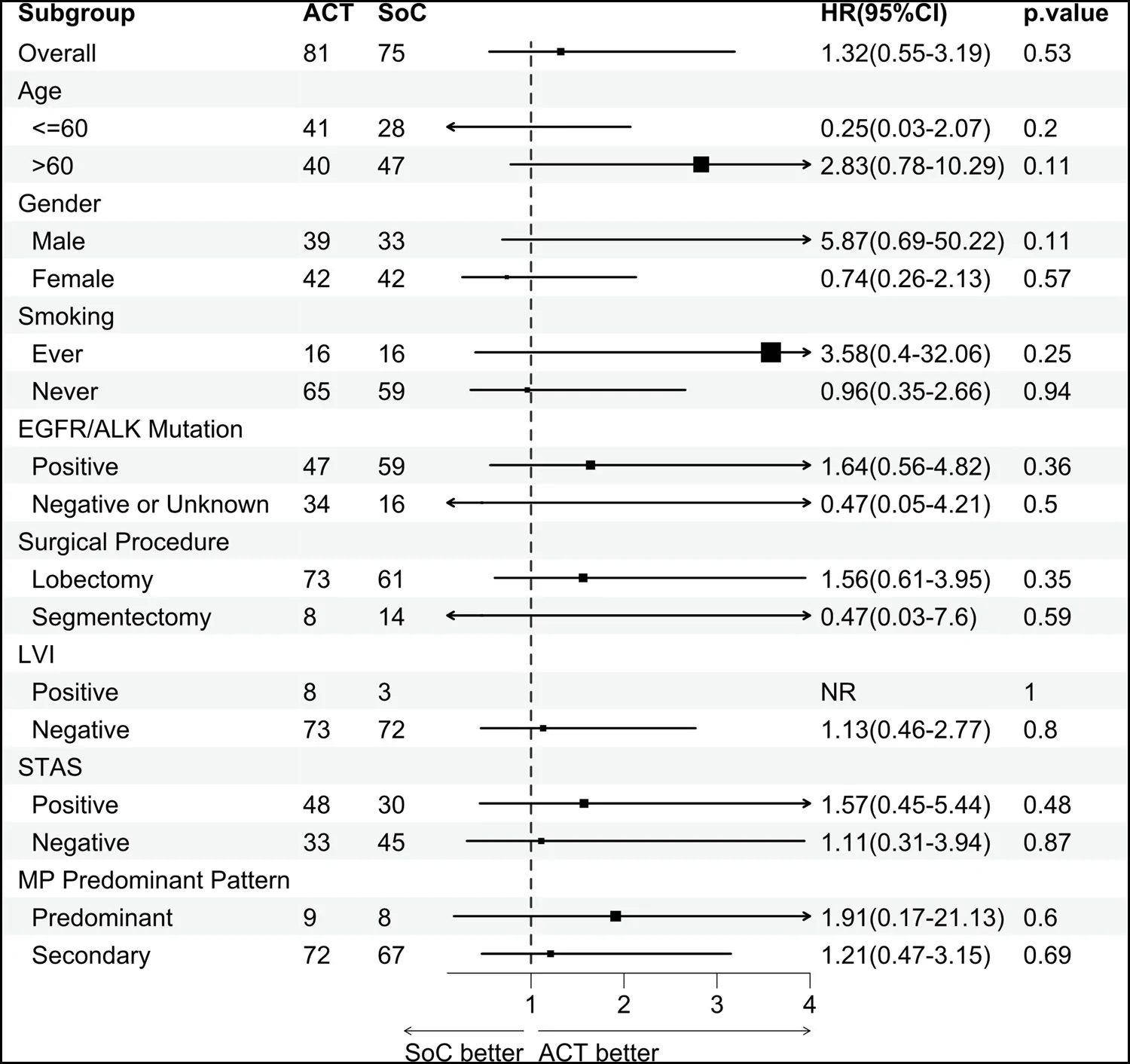
Subgroup Analyses of Recurrence-Free Survival. Hazard ratios, 95% CIs, and *P* values were calculated by use of an unstratified Cox proportional hazard model. Abbreviations: LVI = lymphatic-vascular invasion; MP = micropapillary pattern; STAS = spread through air spaces.

**Table 3.** Subgroup Analysis for Recurrence-Free Survival for All Patients

Variables	ACT		SoC		SoC vs ACT		
	Number	Events	Number	Events	5-year RFS (%)	HR (95% CI)	Log-rank *P*
Age (years)							
≤60	41	6	28	1	85.4 vs 96.4	0.250 (0.030-2.070)	.200
>60	40	3	47	10	95.0 vs 87.2	2.830 (0.778-10.300)	.110
Gender							
Male	39	1	33	5	97.4 vs 81.8	5.870 (0.685-50.200)	.110
Female	42	8	42	6	81.0 vs 85.7	0.740 (0.256-2.130)	.570
Smoking status							
Ever	16	1	16	4	93.8 vs75.0	3.580 (0.400-32.100)	.250
Never	65	8	59	7	87.7 vs 89.8	0.960 (0.349-2.660)	.940
EGFR/ALK mutation							
Positive	47	5	59	10	89.4 vs 84.7	1.640 (0.561-4.820)	.360
Negative or unknown	34	4	16	1	88.2 vs 93.8	0.470 (0.053-4.210)	.500
Surgical procedure							
Lobectomy	73	8	61	10	89.0 vs 85.2	1.560 (0.615-3.950)	.350
Segmentectomy	8	1	14	1	87.5 vs 92.9	0.470 (0.029-7.600)	.590
LVI							
Positive	8	0	3	1	100.0 vs 66.7	NR	/
Negative	73	9	72	10	87.6 vs 87.5	1.130 (0.457-2.770)	.800
STAS							
Positive	48	5	30	5	89.6 vs 86.7	1.570 (0.453-5.440)	.480
Negative	33	4	45	6	87.9 vs 86.7	1.110 (0.314-3.940)	.870
MP pattern							
Predominant (MP++)	9	1	8	2	88.9 vs 75.0	1.910 (0.173-21.10)	.600
Secondary (MP+)	72	8	67	9	88.9 vs 88.1	1.210 (0.467-3.150)	.690

Abbreviations: ACT = adjuvant chemotherapy; CI = confidence interval; HR = hazards ratio; LVI = lymphatic-vascular invasion; MP = micropapillary pattern; SoC = standard of care; STAS = spread through air spaces.

An extended Cox proportional hazards model was performed to further adjust for margin distance and grade and to explore the potential confounding impact on RFS. In the extended model, neither margins nor grade showed a statistically significant association with RFS, which aligns with the initial findings (**Table S3**).

### Safety of adjuvant chemotherapy

Treatment-related AEs (TRAEs) were listed in **Table S4**. TRAEs were observed in 64 patients (79%). Grade 3/4 TRAEs occurred in 13 patients (16.0%). No grade 5 TRAEs were observed in the ACT-Group. Myelosuppression (38.3%), infection and fever (18.5%), pneumonia (17.3%), and hepatic dysfunction (12.3%) were the most common TRAEs during adjuvant AC-chemotherapeutic treatment.

## DISCUSSION

Histopathological MP pattern has been recognized as a high-risk factor for NSCLC recurrence after treatment, even among patients with stage IA disease.^6^^,^^7^^,^^11–13^ For this patient population, the efficacy of ACT remains unclear.^17^ In operable locally-advanced LUADmp patients, resection followed by ACT is recommended as the standard therapeutic regimen, first advocated by Tsao et al^18^ in 2015. In 2018, Qian et al^6^ demonstrated a critical role of ACT in stage IB LUADmp patients. However, the number of studies focused on adjuvant treatment for stage IA LUAD is relatively low, and the outcomes of surgical treated pathological stage IA LUADmp remains poor.^13^

In the current study, there was no significant difference between the 5-year RFS for the ACT-group vs the SoC-group in resected stage IA LUADmp patients, even among patients with MP++ (HR, 1.910; 95% CI, 0.173-21.10; *P* = .600). More importantly, compared with the patients in the SoC-group, 4 cycles of ACT treatment failed to improve the prognosis (5-year RFS) of stage IA LUADmp patients.

In 2016, Yanagawa et al^11^ demonstrated that, after complete resection, the RFS of pathological stage I NSCLC patients with MP was significantly poorer than that of patients without MP (HR, 5.49; 95% CI, 3.09-9.91, *P *< .001). This difference in RFS was not observed between MP++ and MP+ NSCLC patients with stage IA disease postoperatively.^11^^,^^19^ However, in the same year, Tsubokawa et al^13^ demonstrated that, for clinical stage IA LUADmp patients, the proportion of MP pattern did make a difference with MP more than 5% portending a significantly poorer prognosis, especially if the MP proportion was more than 30%.

In the current study, multivariate survival analysis using Cox proportional hazards modelling demonstrated no significant difference in RFS for stage IA LUAD patients with MP+ versus MP++. Importantly, an increasing proportion of the MP component did not correlate with worsening prognosis. There are a few reasons that may explain this phenomenon. First, for stage IA LUADmp, surgical resection is the most crucial factor to predict survival. Second, the median follow-up was 5 years may still not be enough time to reflect differences in prognosis after intervention. Thirdly, we hypothesize that there may be some unrecognized pathological subtypes that may co-exist with MP, and may affect the prognosis of stage IA patients with MP.

Suda et al^20^ found that EGFR mutation status may predict outcomes in early-stage LUAD patients after surgery. Those with EGFR mutations had better RFS (HR 0.894, 95% CI, 0.814-0.980, *P* = .017) and OS (HR 0.729, 95% CI, 0.642-0.829, *P* < .001) than EGFR-negative patients. Additionally, Exon 21-L858R mutations were associated with significantly improved RFS over Exon 19-Del (HR 1.276, 95% CI, 1.107-1.471, *P* = .001).^20^

In our analysis, there was a significant difference in the distribution of driver gene mutation status between the ACT-group and the SoC-group. Compared with patients receiving ACT, the proportion of patients with an *EGFR/ALK* mutation in the SoC-group was significantly higher (78.7% vs 58.0%, *P *= .006). However, based on our survival analysis, driver gene mutation status did not significantly affect the prognosis of surgically-treated LUADmp patients with stage IA disease, and ACT did not improve RFS in patients regardless of their driver gene mutation status. These findings suggest that for pathological stage IA LUADmp patients with driver gene mutation, ACT may not improve prognosis. Larger-scale studies that divide patients according to their driver gene mutation status to evaluate the efficacy of adjuvant treatment will be needed to confirm these results.

In the analysis by Toyokawa et al,^21^ compared with patients without STAS, STAS associated with worse prognosis postoperatively and was a predictive factor of recurrence for NSCLC.

In our study, there was a significant difference in the proportion of patients with STAS between ACT and SoC-group (ACT-group vs SoC-group: 59.3% vs 40.0%, *P *= .016), and STAS could not predict RFS independently (HR: 0.744, *P *= .536). This conclusion was supported by a retrospective analysis conducted by Chen et al,^22^ which demonstrated that for NSCLC without lymph node metastasis (pN0), STAS was not an independent prognostic factor for RFS in patients with LUADmp. These findings demonstrated that for pathological stage IA LUADmp patients with STAS, ACT may not improve prognosis. Effective adjuvant treatment needs to be explored urgently to improve the prognosis of early-stage LUADmp patients.

Our study has several limitations. First, the ACT-group was a prospective cohort while the SoC-group was a retrospective cohort, the bias of baseline characteristics distributed between patients in the ACT-group and SoC-group is inevitable. Meanwhile, some other parameters, including SUVmax, comorbidity burden, performance status, and pulmonary function, that we did not capture, were also attributed to the choice of treatment, causing extra biases. Second, although the sample size included in this study met our study design, it is still a relatively small sample. Third, this design precludes causal inference, and unmeasured confounding may explain the observed similarity in RFS rather than the true absence of an ACT effect.

## CONCLUSIONS

Our results demonstrate that for resected pathological stage IA LUADmp patients, ACT did not significantly improve RFS. While these findings suggest ACT may not be effective for this population, they require verification through prospective phase III trials, alongside further evaluation of how MP pattern proportions and driver gene mutations impact prognosis.

## Supplementary Material

ivag165_Supplementary_Data

## Data Availability

All relevant data are included in the manuscript and its supporting information files.
