# Supplementary material for: Adjuvant Chemotherapy for Resected Stage-IA Lung Adenocarcinoma With Micropapillary Histologic Pattern
Source: Interdiscip Cardiovasc Thorac Surg. 2026 Jun 9;41(10):ivag165. doi: 10.1093/icvts/ivag165 (PMC13685809; doi:10.1093/icvts/ivag165)
Supplement: Supplementary file 1 [file ivag165_supplementary_data.zip › Supplementary_Figure.docx]

Supplementary Figure 1:

The flow diagram of the study. LUADmp, lung adenocarcinoma with micropapillary pattern; ACT, adjuvant chemotherapy; SoC, standard of care.

Supplementary Figure 2:

Recurrence-free survival between the ACT and SoC patients after PSM. ACT, adjuvant chemotherapy; SoC, standard of care; PSM, propensity score matching.

Supplementary Figure 3. Recurrence-free survival according to stage.

(A) Recurrence-Free Survival by Pathologic Stage.
(B) Recurrence-Free Survival in Stage IA1 patients stratified by group
(C) Recurrence-Free Survival in Stage IA2 patients stratified by group
(D) Recurrence-Free Survival in Stage IA3 patients stratified by group
