# Supplementary material for: Adjuvant Chemotherapy for Resected Stage-IA Lung Adenocarcinoma With Micropapillary Histologic Pattern
Source: Interdiscip Cardiovasc Thorac Surg. 2026 Jun 9;41(10):ivag165. doi: 10.1093/icvts/ivag165 (PMC13685809; doi:10.1093/icvts/ivag165)
Supplement: Supplementary file 1 [file ivag165_supplementary_data.zip › Supplemental_Table.docx]

Supplementary Table 1. Univariate and multivariate survival analysis by Cox proportional hazards modeling for all patients, excluding those in the ACT arm who did not complete the full four cycles of treatment (n= 139)

| **Variables** | **Univariate** | | | **Multivariate** | | |
| --- | --- | --- | --- | --- | --- | --- |
|  | **HR** | **95% CI** | ***P*** | **HR** | **95% CI** | ***P*** |
| **Age (years)** |  |  |  |  |  |  |
| ≤ 60 | 1.000 |  |  | 1.000 |  |  |
| > 60 | 1.716 | 0.604-4.872 | 0.310 | 1.877 | 0.636-5.539 | 0.254 |
| **Gender** |  |  |  |  |  |  |
| Male | 1.000 |  |  | 1.000 |  |  |
| Female | 1.623 | 0.600-4.390 | 0.340 | 3.662 | 0.737-18.185 | 0.112 |
| **Smoking Status** |  |  |  |  |  |  |
| Ever | 1.000 |  |  | 1.000 |  |  |
| Never | 0.575 | 0.203-1.633 | 0.299 | 0.247 | 0.045-1.367 | 0.109 |
| **EGFR/ALK Mutation** |  |  |  |  |  |  |
| Positive | 1.000 |  |  | 1.000 |  |  |
| Negative or Unknown | 0.612 | 0.199-1.877 | 0.390 | 0.779 | 0.241-2.514 | 0.676 |
| **Surgical Procedure** |  |  |  |  |  |  |
| Lobectomy | 1.000 |  |  | 1.000 |  |  |
| Segmentectomy | 0.327 | 0.043-2.470 | 0.279 | 0.313 | 0.038-2.574 | 0.280 |
| **LVI** |  |  |  |  |  |  |
| Positive | 1.000 |  |  | 1.000 |  |  |
| Negative | 1.110 | 0.147-8.387 | 0.919 | 1.730 | 0.118-25.413 | 0.689 |
| **STAS** |  |  |  |  |  |  |
| Positive | 1.000 |  |  | 1.000 |  |  |
| Negative | 0.861 | 0.332-2.231 | 0.758 | 0.620 | 0.224-1.720 | 0.358 |
| **MP Pattern** |  |  |  |  |  |  |
| Predominant (MP++) | 1.000 |  |  | 1.000 |  |  |
| Secondary (MP+) | 0.934 | 0.214-4.088 | 0.928 | 0.866 | 0.129-5.811 | 0.882 |
| **Adjuvant Treatment** |  |  |  |  |  |  |
| SoC | 1.000 |  |  | 1.000 |  |  |
| ACT | 0.628 | 0.232-1.700 | 0.360 | 0.643 | 0.225-1.838 | 0.410 |

Abbreviations: LVI, lymphatic-vascular invasion; STAS, Spread through air spaces; MP, micropapillary pattern; ACT, adjuvant chemotherapy; SoC, standard of care; HR, hazards ratio; CI, confidence interval.

Supplementary Table 2. Baseline characteristics of completely resected NSCLC patients with stage IA disease and MP pattern before and after PSM

| **Variables** | **Before PSM, No. of Patient (%)** | | | |  | **After PSM, No. of Patient (%)** | | | | | | | |  |  |
| --- | --- | --- | --- | --- | --- | --- | --- | --- | --- | --- | --- | --- | --- | --- | --- |
|  | **ALL**  **(N = 156)** | **ACT**  **(N = 81)** | **SoC**  **(N = 75)** | ***P*** | | | **ALL**  **(N = 106)** | | **ACT**  **(N = 53)** | **SoC**  **(N = 53)** | | ***P*** | | | |
| **Median Age, years (range)** | 61.5 (31-75) | 60.0 (31-74) | 64.0 (38-75) | 0.019 | | | 62.0 (38-76) | 62.0 (43-73) | | | 62.0 (38-76) | | 0.924 | |  |
| **Gender, number (%)** |  |  |  | 0.604 | | |  |  | | |  | | 0.845 | |  |
| Male | 72 (46.2) | 39 (48.1) | 33 (44.0) |  | | | 46 (43.4) | 24 (45.3) | | | 22 (41.5) | |  | |  |
| Female | 84 (53.8) | 42 (51.9) | 42 (56.0) |  | | | 60 (56.6) | 29 (54.7) | | | 31 (58.5) | |  | |  |
| **Smoking Status, number (%)** |  |  |  | 0.807 | | |  |  | | |  | | 1.000 | |  |
| Ever | 32 (20.5) | 16 (19.8) | 16 (21.3) |  | | | 19 (17.9) | 9 (17.0) | | | 10 (18.9) | |  | |  |
| Never | 124 (79.5) | 65 (80.2) | 59 (78.7) |  | | | 87 (82.1) | 44 (83.0) | | | 43 (81.1) | |  | |  |
| **EGFR/ALK Mutation, number (%)** |  |  |  | 0.006 | | |  |  | | |  | | 0.518 | |  |
| Positive | 106 (67.9) | 47 (58.0) | 59 (78.7) |  | | | 76 (71.7) | 36 (67.9) | | | 40 (75.5) | |  | |  |
| Negative or Unknown | 50 (32.1) | 34 (42.0) | 16 (21.3) |  | | | 30 (28.3) | 17 (32.1) | | | 13 (24.5) | |  | |  |
| **Surgical Procedure, number (%)** |  |  |  | 0.115 | | |  |  | | |  | | 1.000 | |  |
| Lobectomy | 134 (85.9) | 73 (90.1) | 61 (81.3) |  | | | 94 (88.7) | 47 (88.7) | | | 47 (88.7) | |  | |  |
| Segmentectomy | 22 (14.1) | 8 (9.9) | 14 (18.7) |  | | | 12 (11.3) | 6 (11.3) | | | 6 (11.3) | |  | |  |
| **pMTD, mm (range)** | 20.0 (5-30) | 18.0 (8-30) | 20.0 (5-30) | 0.812 | | | 20.0 (5-30) | 20.0 (9-30) | | | 20.0 (5-30) | | 0.775 | |  |
| **LND, No. (range)** | 10.0 (1-27) | 10.5 (1-24) | 10.2 (1-27) | 0.739 | | | 11.0 (1-27) | 10.0 (1-24) | | | 11.0 (1-27) | | 0.345 | |  |
| **Surgical Margins** | 3.0 (0.5-7) | 3.0 (1-7) | 3.0 (0.5-7) | 0.083 | | | 3.0 (1-7) | 3.0 (1-6) | | | 3.0 (2-7) | | 0.915 | |  |
| **LNDS, No, (range)** | 6 (1-9) | 6 (2-8) | 6 (1-9) | 0.332 | | | 6.0 (1-9) | 6.0 (1-8) | | | 6.0 (1-9) | | 0.667 | |  |
| **Histological grade** |  |  |  | 0.053 | | |  |  | | |  | | 1.000 | |  |
| Grade 2 | 76 (48.7) | 46 (56.8) | 30 (40.0) |  | | | 53 (50.0) | 27 (50.9) | | | 26 (49.1) | |  | |  |
| Grade 3 | 80 (51.3) | 35 (43.2) | 45 (60.0) |  | | | 53 (50.0) | 26 (49.1) | | | 27 (50.9) | |  | |  |
| **LVI, number (%)** |  |  |  | 0.214 | | |  |  | | |  | | 1.000 | |  |
| Positive | 11 (7.1) | 8 (9.9) | 3 (4.0) |  | | | 5 (4.7) | 3 (5.7) | | | 2 (3.8) | |  | |  |
| Negative | 145 (92.9) | 73 (90.1) | 72 (96.0) |  | | | 101 (95.3) | 50 (94.3) | | | 51 (96.2) | |  | |  |
| **STAS, number (%)** |  |  |  | 0.016 | | |  |  | | |  | | 0.697 | |  |
| Positive | 78 (50.0) | 48 (59.3) | 30 (40.0) |  | | | 49 (46.2) | 23 (43.4) | | | 26 (49.1) | |  | |  |
| Negative | 78 (50.0) | 33 (40.7) | 45 (60.0) |  | | | 57 (53.8) | 30 (56.6) | | | 27 (50.9) | |  | |  |
| **MP Proportion, % (range)** | 15.0 (5-100) | 10.0 (5-100) | 20.0 (5-80) | 0.548 | | | 10.0 (5-80) | 10.0 (5-80) | | | 10.0 (5-80) | | 0.663 | |  |
| **MP Pattern, number (%)** |  |  |  | 0.929 | | |  |  | | |  | | 1.000 | |  |
| Predominant (MP++) | 17 (10.9) | 9 (11.1) | 8 (10.7) |  | | | 9 (8.5) | 5 (9.4) | | | 4 (7.5) | |  | |  |
| Secondary (MP+) | 139 (89.1) | 72 (88.9) | 67 (89.3) |  | | | 97 (91.5) | 48 (90.6) | | | 49 (92.5) | |  | |  |
| **Prognosis, number (%)** |  |  |  | 0.671 | | |  |  | | |  | | 0.554 | |  |
| Recurrence | 20 (12.8) | 9 (11.1) | 11 (14.6) |  | | | 13 (12.3) | 8 (15.1) | | | 5 (9.4) | |  | |  |
| Non-Recurrence | 136 (87.2) | 72 (88.9) | 64 (85.3) |  | | | 93 (87.7) | 45 (84.9) | | | 48 (90.6) | |  | |  |
| **Prognosis at 5-years, number (%)** |  |  |  | 0.858 | | |  |  | | |  | | 0.358 | |  |
| Recurrence | 19 (12.2) | 9 (11.1) | 10(13.3) |  | | | 12 (11.3) | 8 (15.1) | | | 4 (7.5) | |  | |  |
| Non-Recurrence | 137 (87.8) | 72(88.9) | 65 (86.7) |  | | | 94 (88.7) | 45 (84.9) | | | 49 (92.5) | |  | |  |

Abbreviations: pMTD, pathological maximum tumor dimension; LND, lymph node dissection; LNDS, lymph node dissection station; LVI, lymphatic-vascular invasion; STAS, Spread through air spaces; NSCLC, non-small cell lung cancer; MP, micropapillary pattern; ACT, adjuvant chemotherapy; SoC, standard of care.

Supplementary Table 3. Extended univariate and multivariate survival analysis by Cox proportional hazards modeling for all patients (n = 156).

| **Variables** | **Univariate** | | | **Multivariate** | | | | |
| --- | --- | --- | --- | --- | --- | --- | --- | --- |
|  | **HR** | **95% CI** | ***P*** | | **HR** | **95% CI** | ***P*** |  |
| **Age (years)** |  |  |  | |  |  |  |  |
| ≤ 60 | 1.000 |  |  | | 1.000 |  |  |  |
| > 60 | 1.482 | 0.591-3.717 | 0.401 | | 1.696 | 0.645- 4.464 | 0.285 |  |
| **Gender** |  |  |  | |  |  |  |  |
| Male | 1.000 |  |  | | 1.000 |  |  |  |
| Female | 2.221 | 0.854-5.718 | 0.102 | | 5.450 | 1.067-27.845 | **0.042** |  |
| **Smoking Status** |  |  |  | |  |  |  |  |
| Ever | 1.000 |  |  | | 1.000 |  |  |  |
| Never | 0.830 | 0.301-2.283 | 0.717 | | 0.257 | 0.045- 1.456 | 0.125 |  |
| **EGFR/ALK Mutation** |  |  |  | |  |  |  |  |
| Positive | 1.000 |  |  | | 1.000 |  |  |  |
| Negative or Unknown | 0.670 | 0.244-1.844 | 0.438 | | 0.763 | 0.266 - 2.187 | 0.615 |  |
| **Surgical Procedure** |  |  |  | |  |  |  |  |
| Lobectomy | 1.000 |  |  | | 1.000 |  |  |  |
| Segmentectomy | 0.619 | 0.144-2.674 | 0.521 | | 0.720 | 0.147- 3.515 | 0.685 |  |
| **Margins (cm)** | 0.979 | 0.683-1.405 | 0.910 | | 1.041 | 0.719-1.508 | 0.830 |  |
| **Histological grade** |  |  |  | |  |  |  |  |
| Grade 2 | 1.000 |  |  | | 1.000 |  |  |  |
| Grade 3 | 2.208 | 0.848-5.750 | 0.105 | | 2.169 | 0.782-6.168 | 0.135 |  |
| **LVI** |  |  |  | |  |  |  |  |
| Positive | 1.000 |  |  | | 1.000 |  |  |  |
| Negative | 1.377 | 0.184-10.300 | 0.521 | | 3.582 | 0.333-38.548 | 0.293 |  |
| **STAS** |  |  |  | |  |  |  |  |
| Positive | 1.000 |  |  | | 1.000 |  |  |  |
| Negative | 1.017 | 0.409-2.363 | 0.971 | | 0.833 | 0.323-2.150 | 0.706 |  |
| **MP Pattern** |  |  |  | |  |  |  |  |
| Predominant (MP++) | 1.000 |  |  | | 1.000 |  |  |  |
| Secondary (MP+) | 0.703 | 0.206-2.400 | 0.574 | | 0.679 | 0.152-3.023 | 0.611 |  |
| **Adjuvant Treatment** |  |  |  | |  |  |  |  |
| SoC | 1.000 |  |  | | 1.000 |  |  |  |
| ACT | 0.756 | 0.313-1.827 | 0.535 | | 1.033 | 0.380-2.805 | 0.950 |  |

Abbreviations: LVI, lymphatic-vascular invasion; STAS, Spread through air spaces; MP, micropapillary pattern; ACT, adjuvant chemotherapy; SoC, standard of care; HR, hazards ratio; CI, confidence interval.

Supplementary Table 4. Most common adverse events in the ACT group

| **Type of Event** | **No. of Patients** | |
| --- | --- | --- |
|  | **All Grades (%)** | **Grade ≥ 3 (%)** |
| **Adverse Event** | 64 (79.0) | 13 (16.0) |
| Myelosuppression | 31 (38.3) | 6 (7.4) |
| Infection and Fever | 15 (18.5) | 1 (1.2) |
| Pneumonia | 14 (17.3) | 3 (3.7) |
| Hepatic Dysfunction | 10 (12.3) | 2 (2.4) |
| Diarrhea and Constipation | 8 (9.9) | 1 (1.2) |
| Rash | 8 (9.9) | 1 (1.2) |
| Peripheral Neuritis | 8 (9.9) | 0 (0.0) |
| Renal Dysfunction | 5 (6.2) | 2 (2.4) |
| Fatigue | 5 (6.2) | 0 (0.0) |
